# HOXC10 promotes tumour metastasis by regulating the EMT-related gene Slug in ovarian cancer

**DOI:** 10.18632/aging.103824

**Published:** 2020-09-07

**Authors:** Yulong Peng, Yuanyuan Li, Yimin Li, Anqi Wu, Lili Fan, Wenli Huang, Chunyan Fu, Zhenghao Deng, Kuansong Wang, Yu Zhang, Guang Shu, Gang Yin

**Affiliations:** 1 Department of Pathology, Xiangya Hospital, School of Basic Medical Sciences, Central South University, Changsha, Hunan Province, China; 2 School of Basic Medical Sciences, Central South University, Changsha, Hunan Province, China; 3 Department of Gynecology, Xiangya Hospital, School of Basic Medical Sciences, Central South University, Changsha, Hunan Province, China

**Keywords:** HOXC10, metastasis, miR-222-3p, ovarian cancer, Slug

## Abstract

The mortality rate of ovarian cancer is the highest among gynaecological cancers, primarily due to metastatic symptoms. Recent studies have shown that HOX genes are crucial in tumour progression, but the underlying mechanisms remain unclear. Here, HOXC10 expression was examined in ovarian cancer tissues. The function of HOXC10 in ovarian cancer metastasis was investigated *in vitro*and via intraperitoneal injection *in vivo*. A total of 158 ovarian cancer patients with adequate records were enrolled for analysis. HOXC10 was associated with metastasis and poor prognosis in ovarian cancer. *In vitro*, HOXC10 overexpression promoted ovarian cancer cell migration. Moreover, HOXC10 positively regulated Slug expression, altering the migration ability of cancer cells. Furthermore, our study showed that miR-222-3p was a suppressor of HOXC10. *In vivo*, a decrease in hepatic metastasis was seen in xenograft mice harbouring tumours with stable HOXC10 overexpression after miR-222-3p agomir (an overexpression reagent) injection. This study provides the first evidence that HOXC10 promotes ovarian cancer metastasis by regulating the transcription of the EMT-related gene Slug. Moreover, we found that HOXC10 is regulated by miR-222-3p. These data highlight the crucial role of HOXC10 in enhancing ovarian cancer metastasis and may provide a therapeutic target for ovarian cancer.

## INTRODUCTION

Ovarian cancer (OC) is one of the most lethal gynaecologic malignancies [1]. Its high mortality is primarily due to the diagnosis of most patients during cancer recurrence after metastasis has already occurred [2] and because residual tumours can stimulate metastasis and infiltrative cancer patterns after surgery [3]. Although advances have been made in detection and therapeutic methods for OC [4–6], additional biomarkers for the detection and therapeutic response of OC are highly sought after [7].

Homeobox (HOX) genes are a family of transcription factors, and the entire 39-gene HOX cluster shares identical organization across 4 subclusters—HOXA, HOXB, HOXC and HOXD [8]. HOX genes are aberrantly expressed in a variety of cancers, including OC [9]. Moreover, activation of HOX family members is closely linked to epithelial-mesenchymal transition (EMT) in cancer progression [10–12].

EMT is a complicated cellular programme [13–14]. During carcinogenesis, cancer cells are epithelial-like in the early stage but transform into mesenchymal-like cells as carcinogenesis progresses [15–16]. More recent evidence has shown that EMT enables primary cancer cells to metastasize to distant tissues [17]. Recent studies have indicated that during OC progression, activation of EGF can facilitate EMT programmes by increasing interleukin-6 (IL-6) expression [18] and that STAT4 is a cofactor for EMT induction [19]. Furthermore, Snail and Slug can inhibit p53-mediated apoptosis and are involved in a self-renewal programme [20]. Despite these findings, the mechanism underlying the EMT programme in OC is incompletely understood. In addition, no evidence has demonstrated that the EMT programme is related to HOX genes in OC.

Among the HOX genes, HOXC family members exhibit markedly increased expression in many tumours [21], and several studies have focused on the HOXC10 gene. In breast cancer, oestrogen has been proposed to suppress HOXC10 expression [22]. Moreover, other researchers have proposed that suppressing the function of HOXC10 might be a promising new strategy to overcome chemotherapeutic resistance in breast cancer [23]. In osteosarcoma, silencing HOXC10 significantly inhibits cell proliferation and induces apoptosis [24]. In lung cancer, HOXC10 promotes migration, invasion and adhesion [25]. Moreover, HOXC10 upregulation significantly increases tumour volumes and promotes the migration and invasion of gastric cancer cells [26]. However, the mechanism underlying the abovementioned phenotypes is unclear, and the role of HOXC10 in OC pathogenesis remains largely unexplored.

In this study, we confirmed that HOXC10 overexpression can enhance OC cell metastasis both *in vitro* and *in vivo*. Mechanistically, we identified that HOXC10 promotes OC metastasis by upregulating Slug. We also found that HOXC10 is targeted by miR-222-3p, which was revealed to inhibit Skov3 cell migration in a previous study [27]. Finally, we measured HOXC10 expression in OC patient tissues and analysed the corresponding patient information to determine that HOXC10 is associated with poor prognosis.

## RESULTS

### Overexpression of HOXC10 is associated with poor prognosis in OC patients

**Figure 1:**
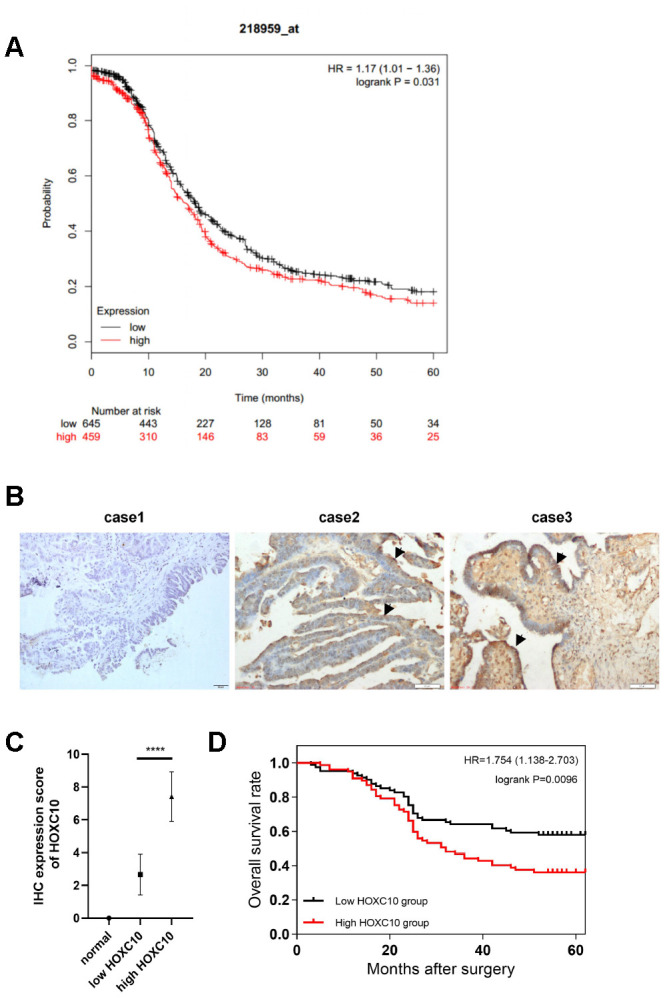
**Overexpression of HOXC10 is associated with poor prognosis in OC patients.** (**A**) Kaplan-Meier survival curves for OC patients (PFS; n = 1207). HR=1.17, P=0.031. (**B**) HOXC10 protein expression in normal ovarian tissues (case 1) and OC patient tissues (case 2, low expression of HOXC10; case 3, high expression of HOXC10) was assessed by IHC staining. Scale bars, 50 μm. (**C**) IHC positive rate scores for HOXC10 in normal tissues and each group of OC patient tissues. P<0.0001. (**D**) Kaplan-Meier survival curves for 158 OC patients. HR=1.754, P=0.0096.

To investigate whether HOXC10 might be associated with the prognosis of OC patients, we analysed the relationship between HOXC10 gene expression and the survival time of OC patients by integrating TCGA database data obtained from the Kaplan-Meier Plotter website (http://kmplot.com/private/). The results of the prognostic analysis for progression-free survival (PFS) are shown in Figure 1A. The prognostic analysis showed that high HOXC10 expression is associated with poor prognosis in OC (HR=1.17, P=0.036).

To confirm the prognostic analysis results, we performed immunohistochemical (IHC) staining on tumour tissue sections from 158 patients diagnosed with OC at Xiangya Hospital between March 2010 and July 2015 and 10 paraffin-embedded normal ovarian tissue sections (Figure 1B). The positive rates of IHC staining for HOXC10 in normal tissues and OC patient tumour tissues are shown in Figure 1C. The clinicopathological characteristics and semiquantitative IHC results are shown in Table 1. Then, the 158 patients were divided into two groups according to their relative expression level of HOXC10 (high, IHC positive rate score>5; low, IHC positive rate score<5). Increased HOXC10 expression was significantly associated with the FIGO stage (P=0.0271), the tumour distant metastasis (P=0.0220) and the survival status at the time of analysis (P=0.0249). However, no significant correlation was found between age (P=0.3521) and histological type (P=0.2685). The Kaplan-Meier curve for the 158 EOC patients showed that the survival times of patients with low HOXC10 expression were significantly longer than those of patients with high HOXC10 expression (mean overall survival time: 45 months vs. 37 months; P = 0.0096; Figure 1D).

**Table 1:** Clinicopathological characteristics and correlations with the HOXC10-based classification (N=158).

**Characteristics**	**Cases**		**HOXC10 expression level**		***P value*3**
	**n**	**%**	**low**	**high**	
**Age (year)**					
< 50	72	54.43%	34	38	0.3521
≥ 50	86	45.57%	47	39	
**Histologic type**					
SC^1^	77	48.73%	36	41	0.2685
Nonserous	81	51.27%	45	36	
**FIGO^2^*stage***					
I	33	20.89%	21	12	0.0271*
II	38	24.05%	24	14	
III	51	32.28%	24	27	
IV	36	22.78%	12	24	
**Tumor distant metastasis**					
Presence	36	22.78%	12	24	0.0220*
Absence	122	77.22%	69	53	
**Survival state**					
Dead	71	44.94%	29	42	0.0249*
Alive	87	55.06%	52	35	

^1^SC, Serous Cancer. ^2^ FIGO, International Federation of Obstetricians and Gynecologists. ^3^ P values ≤ 0.05 were considered significant according to the Fisher's exact test of clinicopathological characteristics for HOXC10.

A Cox regression analysis was further to evaluate and analyze the potential of HOXC10 as a prognostic biomarker in ovarian cancer (Supplementary Table 1). Univariate survival showed that age (P=0.077) and histologic type (P=0.062) were not concerned with ovarian cancer overall survival, and FIGO stage (P=0.011), survival state (P=0.004), distant metastasis (P=0.013) and HOXC10 expression level (P=0.005) were associated with overall survival. In the multivariate Cox regression analysis, FIGO stage (P=0.03, HR=0.663, 95%CI 0.458 to 0.961), survival state (P=0.009, HR=0.557, 95%CI 0.358 to 0.865), distant metastasis (P=0.018, HR=0.639, 95%CI 0.442 to 0.926) and HOXC10 expression level (P=0.008, HR=0.516, 95%CI 0.315 to 0.843) were associated with overall survival. In general, these results suggest that high HOXC10 expression can predict poor prognosis in OC patients.

### HOXC10 accelerates OC cell migration

To investigate the role of HOXC10 in OC, we first measured its expression in OC cell lines. HOXC10 expression was higher in OC cells than in ovarian epithelial cells and was even higher in HO8910PM (PM) cells than in HO8910 (8910) cells (Figure 2A, 2B). Interestingly, 8910 and PM are isogenic cell lines, and the migration ability of PM cells is higher than that of 8910 cells (Figure 2C). We believe that the different migration abilities of these two cell lines are associated with HOXC10.

**Figure 2:**
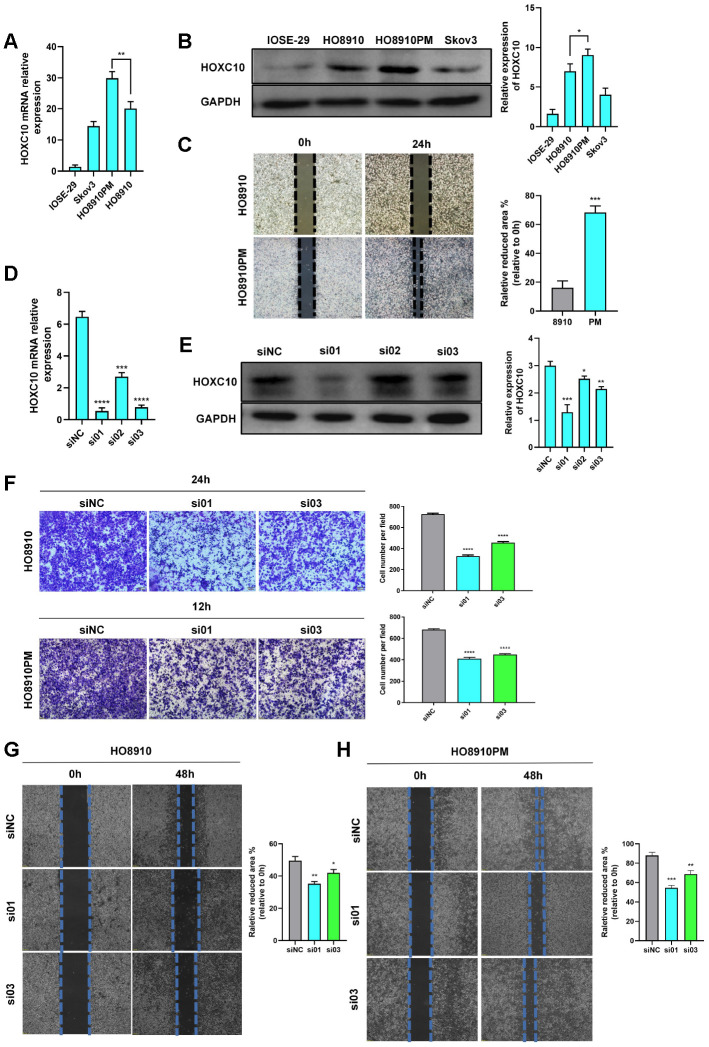
**HOXC10 accelerates OC cell migration.** (**A**, **B**) Relative mRNA and protein expression levels of HOXC10. 8910 vs. PM cells. P=0.0177 (mRNA), P=0.0301 (protein). (**C**) Comparison of the migration ability of 8910 and PM cells via wound healing assays. P=0.0002. Scale bars, 200 μm. (**D**, **E**) Transfection efficiencies of the HOXC10 siRNA products. P<0.0001, P=0.0001 and P<0.0001; P=0.0008, P=0.0120 and P=0.0013. (**F**) Transwell assay of 8910 and PM cells transfected with HOXC10 siRNA products No. 1 and No. 3 and the negative control siRNA. 8910 cell graphs, P<0.0001 and P<0.0001. PM cell graphs, P<0.0001 and P<0.0001. Scale bars, 100 μm. (**G**, **H**) Wound healing assay of 8910 and PM cells transfected with HOXC10 siRNA products No. 1 and No. 3 and the negative control siRNA. P=0.0010 and P=0.0167; P=0.0025 and P=0.0097. Scale bars, 200 μm.

**Figure 2:**
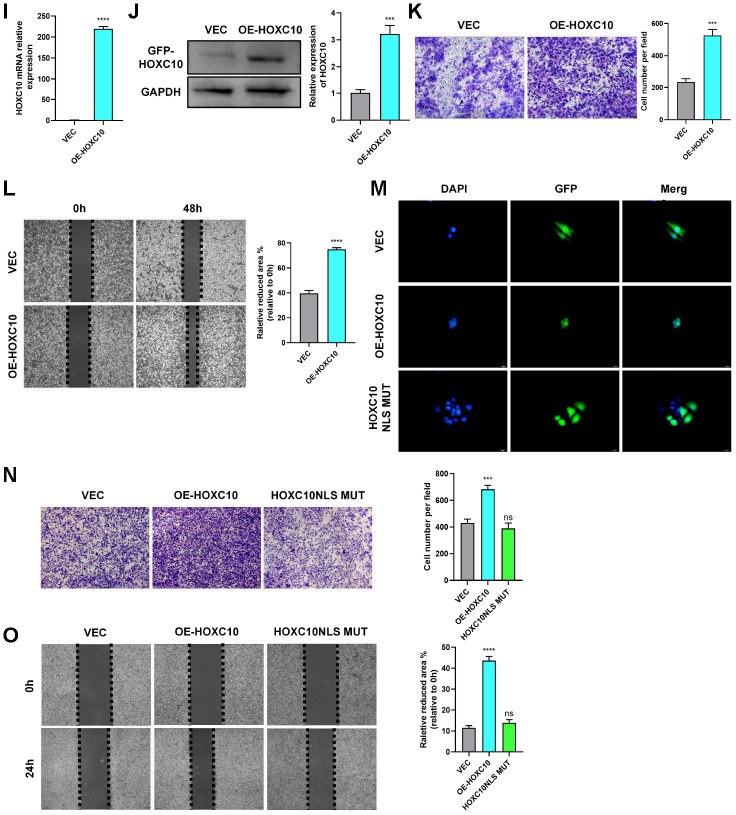
**HOXC10 accelerates OC cell migration.** (**I**, **J**) Relative mRNA and protein expression levels of HOXC10 in 8910 cells transfected with the HOXC10 overexpression plasmid and empty vector. P<0.0001, P=0.0003. (**K**, **L**) Transwell and wound healing assays of Skov3 cells transfected with the HOXC10 overexpression plasmid and empty vector. P=0.0003, P<0.0001. Scale bars, 100 μm and 200 μm, respectively. (**M**) Fluorescence signal in 8910 cells transfected with the HOXC10 overexpression plasmid, HOXC10 NLS mutation plasmid and empty vector; all cells were stained with DAPI. Scale bars, 25 μm. (**N**, **O**) Transwell and wound healing assays of 8910 cells transfected with the HOXC10 overexpression plasmid, HOXC10 NLS mutation plasmid and empty vector. P=0.0005 and P=0.2381; P<0.0001 and P=0.0827. Scale bars, 100 μm and 200 μm, respectively.

We purchased siRNA products targeting the HOXC10 gene (siHOXC10) and measured their transfection efficiencies (Figure 2D, 2E). The No. 1 and No. 3 siRNA products were used in follow-up experiments. We performed transwell and wound healing assays using PM and 8910 cell lines transfected with siHOXC10 No. 1 and No. 3 and a negative control siRNA. In both cell lines, cells in the knockdown experimental groups migrated slower than those in the corresponding negative control group, indicating that the cell migration ability was suppressed when HOXC10 was downregulated in these cell lines (Figure 2F–2H).

We next constructed an HOXC10 overexpression plasmid and evaluated its overexpression efficiency (Figure 2I, 2J). Via inverted fluorescence microscopy, we observed that the fluorescence signal for the HOXC10-GFP fusion protein was localized in the nucleus, whereas the fluorescence signal for the empty vector was localized throughout the cell (Figure 2M). To further verify these results, we stained the cells with DAPI and performed analysis with a high-content imaging system. Blue fluorescence and green fluorescence were completely colocalized only in the HOXC10 overexpression groups (Figure 2M). We then overexpressed HOXC10 in the Skov3 cell line, which exhibited the lowest endogenous expression level of HOXC10 among the tested cell lines, and found in transwell and wound healing assays that the migration ability of these cells was enhanced (Figure 2K, 2L). And cell proliferation assay showed that cell proliferative ability was not enhanced by HOXC10 (Supplementary Figure 4). Furthermore, we mutated the nuclear localization signal (NLS) sequence of HOXC10 to compare the effects of this mutation with those of HOXC10 overexpression. Interestingly, after the HOXC10 NLS was mutated, the fluorescence signal from the HOXC10-GFP fusion protein became localized throughout the cell (Figure 2M). Then, we performed cell immunofluorescence staining to verify this phenomenon (Supplementary Figure 3). Moreover, HOXC10 overexpression promoted OC cell migration, but the increased migration ability was abolished when the HOXC10 NLS was mutated (Figure 2N, 2O). The above results clarified that the effect of HOXC10 on OC cell migration is based on its endonuclear localization.

### HOXC10 promotes OC cell migration by regulating Slug transcription

To study the mechanism by which HOXC10 promotes OC cell migration, we analysed HOXC10 expression through gene set enrichment analysis (GSEA) using TCGA profiles. The HOXC10 expression level was positively correlated with the TGF-β and FAK pathway activity (Figure 3A). The EMT programme is closely related to the TGF-β [28] and FAK pathways [29] and has been shown to greatly affect tumour metastasis [17]. Therefore, we measured the mRNA expression levels of EMT-related genes upon HOXC10 downregulation and upregulation. The quantitative PCR (qPCR) data showed that the mRNA expression level of Slug was positively correlated with that of HOXC10 (Figure 3B, 3C). Then we checked other EMT-related epithelial genes. We found Claudin-3 and MUC15 were negatively regulated by HOXC10 (Supplementary Figure 7).

**Figure 3:**
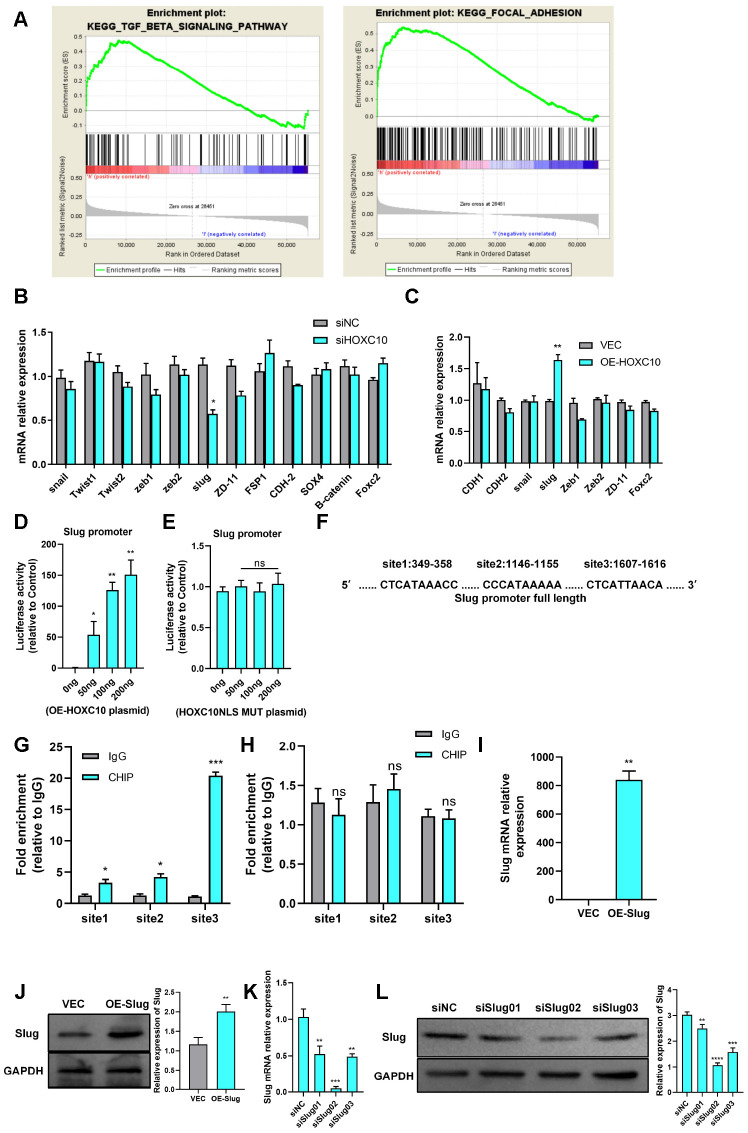
**HOXC10 promotes OC cell migration by regulating Slug transcription.** (**A**) The GSEA plot indicates that HOXC10 expression is positively correlated with TGF-β and FAK pathway signatures (OC sample data were downloaded from TCGA, n=379). (**B**, **C**) mRNA expression of EMT-related genes in 8910 cells transfected with HOXC10 siRNA, negative control siRNA, the HOXC10 overexpression plasmid, and empty vector. P=0.0257 and P=0.0093. (**D**) 8910 cells were cotransfected with a plasmid containing the full-length Slug promoter and increasing concentrations of the HOXC10 overexpression plasmid. P=0.0499, P=0.0032 and P=0.0079. (**E**) 8910 cells were cotransfected with the plasmid containing the full-length Slug promoter and increasing concentrations of the HOXC10 NLS mutation plasmid. P=0.1005, P=0.9559 and P=0.4059. (**F**) Schematic diagram of three predicted HOXC10 binding sites in the Slug promoter region. (**G**) Relative fold enrichment for IgG at the three predicted binding sites, as evaluated by ChIP-qPCR. P=0.0162, P=0.0158 and P=0.0003. (**H**) Relative fold enrichment for IgG at the three predicted binding sites after deletion of the HOXC10 DNA binding sites. P=0.5259, P=0.3579 and P=0.8293. (**I**, **J**) Relative mRNA and protein expression levels of Slug in 8910 cells transfected with the Slug overexpression plasmid and empty vector. P=0.0018, P=0.0044. (**K**–**L**) Transfection efficiencies of the Slug siRNA products. P=0.0048, P=0.0001, and P=0.0013; P=0.0083, P<0.0001, and P=0.0002.

**Figure 3:**
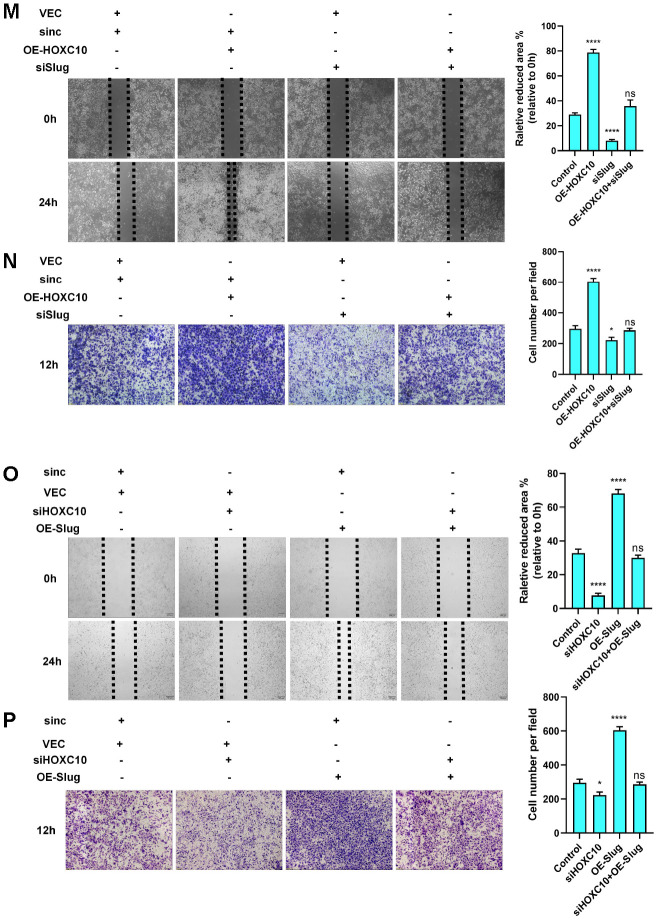
**HOXC10 promotes OC cell migration by regulating Slug transcription.** (**M**, **N**) Rescue experiment using 8910 cells cotransfected with the HOXC10 overexpression plasmid, siRNA targeting Slug, empty vector or negative control. P<0.0001, P<0.0001, and P=0.0832; P<0.0001, P=0.0107, and P=0.5562. Scale bars, 200 μm and 100 μm, respectively. (**O**, **P**) Rescue experiment using 8910 cells cotransfected with siRNA targeting HOXC10, the Slug overexpression plasmid, negative control or empty vector. P<0.0001, P<0.0001, and P=0.1647; P<0.0001, P=0.0107, and P=0.5562. Scale bars, 200 μm and 100 μm, respectively.

Slug is a crucial regulator of EMT. To determine the precise relationship between HOXC10 and Slug, we first constructed a Slug promoter plasmid and then performed a luciferase reporter gene assay. As shown in the figures, the fluorescence activity of the Slug promoter increased as the concentration of the HOXC10-overexpressing plasmid increased (Figure 3D), but the HOXC10 NLS mutation had no effect on this activity (Figure 3E). For further confirmation, we performed chromatin immunoprecipitation (ChIP) with an anti-HOXC10 antibody. Primers specific for three predicted Slug binding site sequences were designed (Figure 3F), and sequence No. 3 was found to have the highest binding ability (Figure 3G). After the HOXC10 DNA binding site was deleted, the binding ability of HOXC10 to Slug was abolished (Figure 3H). The above results confirmed that Slug is a downstream gene of HOXC10. After evaluating the overexpression efficiency of the Slug overexpression plasmid (Figure 3I-J) and the knockdown efficiency of the Slug siRNA product (Figure 3K, 3L), we performed a rescue experiment. Downregulation of Slug expression reversed the promotive effect of HOXC10 overexpression on OC cell migration (Figure 3M, 3N). In contrast, Slug overexpression reversed the reduction in cell migration induced by HOXC10 silencing (Figure 3O, 3P). The Slug protein expression levels after rescue were shown in Supplementary Figure 5. These data indicated that HOXC10 promotes OC cell migration by regulating Slug and subsequently affecting the EMT programme.

### HOXC10 expression is regulated by miR-222-3p

MicroRNAs (miRNAs) can act as either tumour suppressors or tumour promoters by repressing the transcription of their target genes [30]. Thus, we used three miRNA binding site prediction websites to identify the potential miRNAs targeting HOXC10. We subsequently focused on one of the overlapping miRNAs, miR-222-3p, because a previous study demonstrated that miR-222-3p can inhibit OC cell proliferation and migration (Figure 4A) [27]. The candidate microRNAs from three prediction websites were listed in Supplementary Table 2.

**Figure 4:**
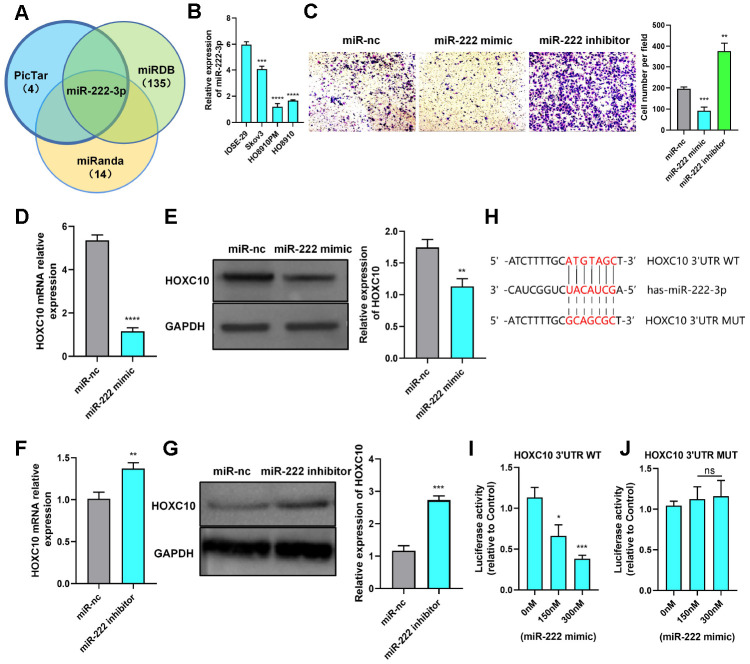
**HOXC10 expression is regulated by miR-222-3p.** (**A**) A Venn diagram was used to identify the candidate miRNAs targeting HOXC10. (**B**) Relative expression of miR-222-3p in cell lines. P=0.0004, P<0.0001, and P<0.0001. (**C**) Transwell assay of PM cells transfected with the miR-222-3p mimic, miR-222-3p inhibitor or negative control. P=0.0007 and P=0.0012. Scale bars, 100 μm. (**D**, **E**) Relative mRNA and protein expression levels of HOXC10 in PM cells transfected with the miR-222-3p mimic or negative control. P<0.0001 and P=0.0037. (**F**, **G**) Relative mRNA and protein expression levels of HOXC10 in Skov3 cells transfected with the miR-222-3p inhibitor or negative control. P=0.0041 and P=0.0002. (**H**) Schematic diagram of the binding site for miR-222-3p in the HOXC10 3’UTR. (**I**) PM cells were cotransfected with a plasmid containing the full-length HOXC10 3’UTR and increasing concentrations of the miR-222-3p mimic. P=0.0108 and P=0.0005. (**J**) PM cells were cotransfected with a plasmid containing the mutated full-length HOXC10 3’UTR and increasing concentrations of the miR-222-3p mimic. P=0.4474 and P=0.3730.

**Figure 4:**
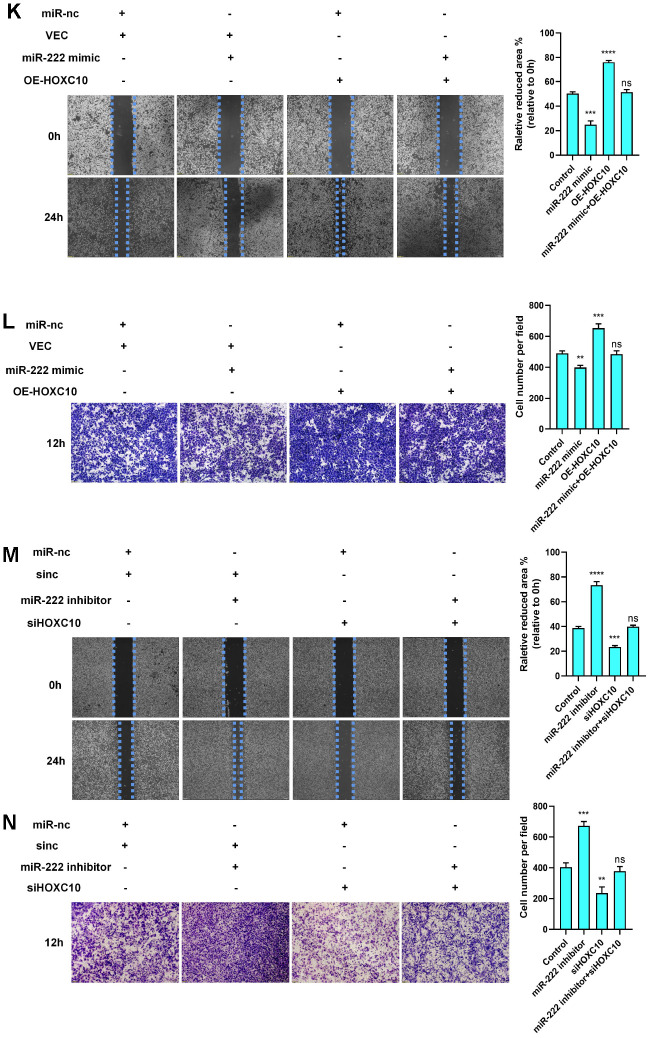
**HOXC10 expression is regulated by miR-222-3p.** (**K**, **L**) Rescue experiment with 8910 cells cotransfected with the miR-222-3p mimic, HOXC10 overexpression plasmid, miR negative control or empty vector. P=0.0002, P<0.0001, and P=0.4823; P=0.0017, P=0.0010, and P=0.7193. Scale bars, 200 μm and 100 μm, respectively. (**M**, **N**) Rescue experiment with 8910 cells cotransfected with the miR-222-3p inhibitor, siRNA targeting HOXC10, miR negative control or siRNA negative control. P<0.0001, P=0.0001, and P=0.3069; P=0.0003, P=0.0042, and P=0.3539. Scale bars, 200 μm and 100 μm, respectively.

We first measured the expression of miR-222-3p in cell lines and found it to be lower in OC cells than in ovarian epithelial cells (Figure 4B). Then, we reconfirmed that miR-222-3p can inhibit OC cell migration (Figure 4C). In addition, miR-222-3p negatively regulated HOXC10 expression (Figure 4D–4G). To validate the relationship between miR-222-3p and HOXC10, we constructed a luciferase plasmid containing the HOXC10 3’-UTR (Figure 4H). The luciferase reporter assay results showed that the fluorescence activity driven by the HOXC10 3’-UTR plasmid decreased as the miR-222-3p concentration increased (Figure 4I). However, the difference in the fluorescence activity was statistically insignificant when the miR-222-3p binding site was mutated (Figure 4J). These data indicated that miR-222-3p is an upstream regulator of HOXC10 and suppresses HOXC10 expression. Furthermore, a rescue experiment was performed, and the miR-222-3p-mediated reduction in the migration ability of OC cells was reversed by negative regulation of HOXC10 expression (Figure 4K–4N). The HOXC10 protein expression levels after rescue were shown in Supplementary Figure 2.

### HOXC10 promotes OC metastasis *in vivo*, and miR-222-3p can inhibit this process

**Figure 5:**
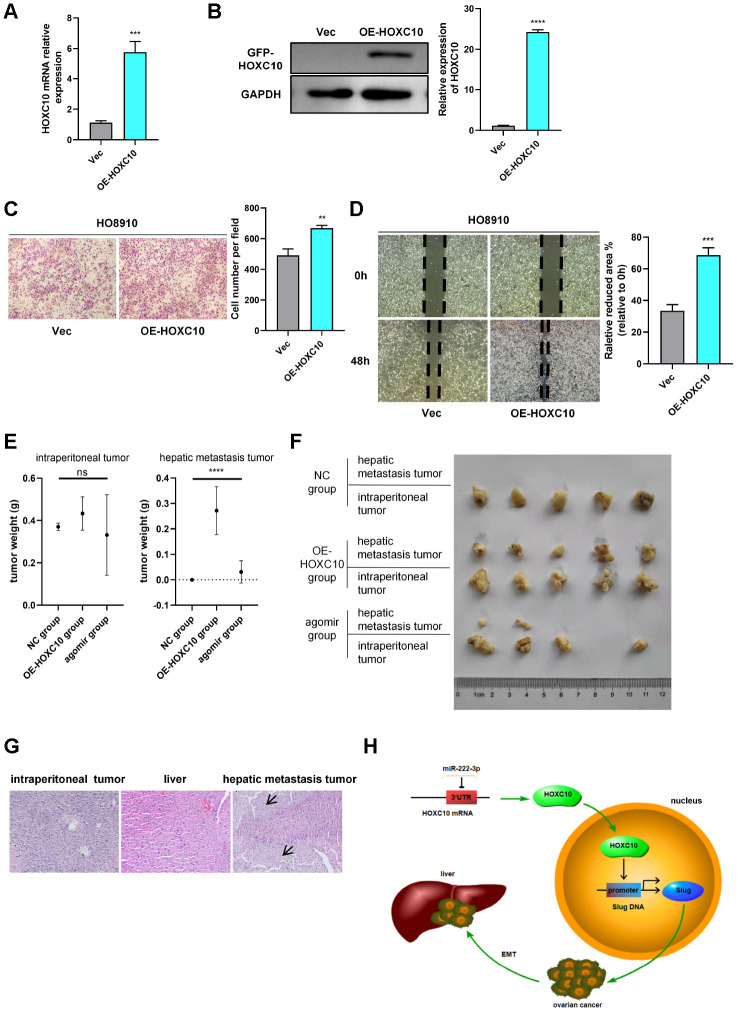
**HOXC10 promotes OC metastasis *in vivo*.** (**A**, **B**) Relative mRNA and protein expression levels of HOXC10 in VEC and OE-HOXC10 cells. P=0.0003 and P<0.0001. (**C**, **D**) Transwell and wound healing assays of VEC and OE-HOXC10 cells. P=0.0027 and P=0.0005. Scale bars, 100 μm and 200 μm, respectively. (**E**) Weights of intraperitoneal tumours and hepatic metastasis tumours from mice in the NC, OE-HOXC10 and agomir groups. (**F**) Photograph of tumours excised from mice. (**G**) HE staining of intraperitoneal tumours and livers from mice in the NC, OE-HOXC10 and agomir groups. The black arrows show the regions of tumour metastasis in the livers. Scale bars, 50 μm. (**H**) A schematic showing that HOXC10 upregulates EMT by directly targeting the downstream Slug gene and that miR-222-3p downregulates HOXC10 by directly binding to its 3’-UTR.

To investigate the functions of HOXC10 *in vivo*, we cultured and screened 8910 cells stably overexpressing HOXC10 and vector cells, named OE-HOXC10 cells and VEC cells, respectively, with G418. Then, we validated the mRNA and protein expression levels of HOXC10 via qPCR and WB (Figure 5A, 5B) and evaluated the biological function of HOXC10 via transwell and wound healing assays (Figure 5C, 5D).

For the tumourigenicity assay, fifteen female athymic nude mice were divided into three groups: the OE-HOXC10 group, the NC group and the agomir group. After the mice were dissected and abdominal tumour outgrowth and metastasis were examined (Supplementary Figure 1), the tumour weights were measured and analysed (Figure 5E). Abdominal tumours were formed in all but one mouse in the agomir group, but non-hepatic metastases were detected only in the NC group (Figure 5F). As shown in Figure 5E, the tumour weights of hepatic metastases differed significantly (P<0.0001) among the three groups. However, without considering hepatic metastases, the primary intraperitoneal tumour weights did not differ significantly among the three groups (P=0.3203). Compared to the livers of mice in the NC group (which exhibited only normal hepatic tissue structure), the livers of all mice in the OE-HOXC10 group and most mice in the agomir group exhibited many invasive tumour lesions (Figure 5G). After testing the mRNA expression of HOXC10 in three mice groups (Supplementary Figure 6), we found that miR-222-3p suppressed the expression of HOXC10 *in vivo*. In summary, upregulation of HOXC10 expression increased ovarian tumour metastasis, and this effect was abrogated by miR-222-3p *in vivo*.

## DISCUSSION

In the current study, we showed that HOXC10 can upregulate Slug expression and that its own expression is regulated by miR-222-3p (Figure 5H). HOXC10 could thus help facilitate OC metastasis. Furthermore, our data showed a direct correlation between HOXC10 expression and the prognosis of OC patients.

In the past few decades, OC has been the leading cause of death from gynaecological cancers [31]. OC is characterized by peritoneal metastasis, rapid proliferation and intraperitoneal malignant ascites [32]. Its nonspecific symptoms result in its high mortality rate [33, 34]. Previous studies have identified many factors potentially associated with the poor prognosis of OC [35–38]. In this study, we found that HOXC10 is associated with the poor prognosis of OC patients.

EMT is consistently correlated with tumour metastasis [39]. We considered the crucial function of EMT genes in OC and found that the Slug gene is positively regulated by HOXC10. Many previous studies have reported that Slug mediates OC metastasis [40–42]. We confirmed that HOXC10 can bind to the Slug promoter and regulate its transcription. These data suggested that HOXC10 can promote OC cell migration via Slug. This mechanism was confirmed by cell rescue experiments. Importantly, this study is the first to find that the HOXC10 gene can positively regulate the Slug gene by regulating cancer metastasis.

Peritoneal metastasis occurs more frequently in OC than in other gynaecological cancers because the peritoneal environment of primary OC has no anatomical barriers [43]. In addition, some HOX genes, such as HOXA4 and HOXB3, could be biomarkers for OC [44, 45]. HOX genes are a family of homeodomain transcription factors [46]. Most studies on HOX genes have focused on their functions, and HOX genes have been reported to promote cell proliferation, differentiation and expansion [47–51]. HOX genes are crucially important for the growth and differentiation of species, and their dysregulation is related to ovarian carcinogenesis [52]. Previous studies have shown that overexpression of different HOX genes leads to different histological subtypes of cancer [53]. Furthermore, HOXA7 mediates the malignant transformation of OC [54]. Overexpression of either HOXB7 or HOXB13 facilitates the proliferation and invasion of OC cells [55, 56], and HOXC13 plays a crucial role in DNA replication [57]. In this study, we demonstrated that HOXC10 overexpression enhances the migration ability of OC cells. Subsequently, we discovered that migration is enhanced via nuclear import of HOXC10 and that this HOXC10-induced enhancement in the migration ability is abolished by HOXC10 NLS mutation. However, the mechanism underlying the nuclear import of HOXC10 remains unclear.

Many miRNAs have been reported to play important roles in OC, generally by repressing their target genes [58–60]. In our previous study, we found that miR-222-3p inhibits cell proliferation and migration in OC [27]. In this study, we comprehensively analysed the HOXC10 binding site prediction data from three websites. We subsequently focused on miR-222-3p, which was previously shown to suppress OC progression. The fluorescent activity of the HOXC10 3’-UTR was inversely correlated with miR-222-3p expression, while the accompanying phenomena were abolished when the binding site sequence was mutated. In addition, miR-222-3p successfully rescued the changes in biological behaviour due to modulation of HOXC10 expression. Thus, we inferred that miR-222-3p acts upstream of HOXC10 and that its ability to suppress OC cell migration is mediated via HOXC10 downregulation.

Our data confirmed that HOXC10 is a crucial regulator in OC and is associated with poor prognosis in patients with OC. HOXC10 promoted OC metastasis by positively regulating Slug transcription. In addition, HOXC10 was negatively regulated by miR-222-3p (Figure 5H). However, HOXC10 influenced tumour metastasis but not cell proliferation in OC, as demonstrated both *in vitro* and *in vivo*. Therefore, enhanced expression of HOXC10 may contribute to enhanced poor prognosis in OC, and miR-222-3p could suppress HOXC10-induced OC metastasis.

## MATERIAL S AND METHODS

### Cell culture

The OC cell lines 8910 (a serous cystadenocarcinoma cell line), PM (a serous cystadenocarcinoma cell line; highly invasive HO8910 cells), Skov3 (a serous papillary cystadenocarcinoma cell line), and IOSE-29 (a human ovarian epithelial cell line) were maintained by the laboratory of Professor Gang Yin (Changsha, China). PM cells were cultured in Dulbecco’s modified Eagle’s medium (DMEM), while the other ovarian cell lines were maintained in RPMI-1640 medium. These two media were supplemented with 10% foetal bovine serum (FBS) (Gibco, Carlsbad, CA). Cells with stably modulated expression were maintained in RPMI-1640 medium supplemented with 10% FBS and G418. All cells were cultured in a humidified 5% CO_2_ incubator at 37°C.

### Plasmid construction

The pEGFP-C1 vector (maintained by the laboratory of Professor Gang Yin) was used to construct the HOXC10 overexpression plasmid. Purified HOXC10 cDNA fragments were digested and were then ligated with T4 DNA ligase (Tsingke, Beijing, China). Next, the above plasmids were transformed into DH5α competent cells (Tsingke). For construction of the HOXC10 NLS mutation plasmid, the HOXC10 CDS was analysed with cNLS Mapper (http://nls-mapper.iab.keio.ac.jp/cgi-bin/NLS_Mapper_form.cgi). Then, we used a ClonExpress II One Step Cloning Kit (Vazyme, Nanjing, China) to subclone sequences into the HOXC10 overexpression plasmid. To construct the wild-type and mutant HOXC10 3’-UTR plasmids, the full-length 3’-UTR of HOXC10, which contained the predicted binding site (sequence: ATGTAGC), was cloned into the psiCHECK-2 vector (HOXC10 3’UTR WT). Then, the binding site sequence was replaced with a mutated sequence (GCAGCGC) via mutagenesis to generate the HOXC10 3’-UTR mutation plasmid (HOXC10 3’UTR MUT). To construct the HOXC10 DNA binding site deletion plasmid, the HOXC10 DNA binding site sequences were evaluated at the NCBI website (https://www.ncbi.nlm.nih.gov/). Then, we used the ClonExpress II One Step Cloning Kit to construct the HOXC10 overexpression plasmid by recombination to delete these sequences. To construct the Slug promoter plasmid, the binding sites of HOXC10 and the Slug promoter were predicted with JASPAR version 5.0 (http://jaspardev.genereg.net/). The full-length promoter of Slug was cloned into the pGL3-Basic vector. The procedure used for *Escherichia coli* culture was the same as that previously described [27].

### Transfection of mature miRNAs, siRNAs and plasmids

We used the following reagents: miR-222-3p mimic, miR-222-3p inhibitor, miR negative control, siRNAs targeting HOXC10, siRNAs targeting Slug and siRNA negative control (sinc). All reagents were purchased from Guangzhou RiboBio Co., Ltd (assay IDs: miR10000279, miR20000279, miR01101, siRNAPack_|1999_HOXC10, siRNAPack_1999_Slug, and siNO5815122147, respectively). The Slug overexpression plasmid was kindly provided by the laboratory of Ceshi Chen (Kunming Institute of Zoology, Kunming, China). miRNAs, siRNAs and plasmids were preincubated with Lipofectamine RNAi Max transfection reagent (Invitrogen) diluted in Opti-MEM (Invitrogen) and were then incubated with Lipofectamine 3000 reagent (Invitrogen) in Opti-MEM for transfection into cells.

### RNA extraction and real-time quantitative PCR

Total RNA was extracted with TRIzol reagent (Invitrogen, CA). Reverse transcription was performed with a GoScript Reverse Transcription System (Promega, Madison, WI, USA). Real-time qPCR with GoTaq qPCR Master Mix (Promega, Madison, WI, USA) in an Applied Biosystems 7500 Real-Time PCR System. The following primers were used: HOXC10, 5’-AAGCGAAAGAGGAGATAAAGGC-3’ (forward) and 5’-GTCTTGCTAATCTCCAGGCGG-3’ (reverse); Slug, 5’-AAGCCAAACTACAGCGAACT-3’ (forward) and 5’-GGTATGACAGGCATGGAGTAA-3’ (reverse); and GAPDH, 5’-GCACCGTCAAGGCTGAGAAC-3’ (forward) and 5’-TGGTGAAGACGCCAGTGGA-3’ (reverse).

### Western blot analysis

The protocols used for cellular protein extraction and Western blotting were the same as those previously described [27]. Western blotting was performed with a polyclonal anti-HOXC10 antibody (1:1000 dilution; ab153904; Abcam) and a polyclonal anti-Slug antibody (1:1000 dilution; ab51772; Abcam). GAPDH (antibody: 1:1000 dilution; 2118; Cell Signaling) was used as the internal control protein.

### Transwell assay and wound healing assay

For the transwell assay, cells (1×10^5^ cells/μl) were resuspended in 200 μl of RPMI-1640 medium or DMEM and seeded in the upper chambers of transwell inserts (8 μm pore size, 24-well plates, Corning). The lower chambers were filled with 750 μl of RPMI-1640 medium or DMEM containing 10% FBS. After incubation, the migrated cells were fixed with 4% paraformaldehyde and stained with 0.1% crystal violet at room temperature. The migrated cells were imaged with a microscope (Olympus Corp., Tokyo, Japan). For the wound healing assay, treated cells were seeded into 6-well plates and incubated. A scratch was made in the confluent cell monolayer to create a cell-free zone, and detached cells were removed. Subsequently, the cells were cultured in serum-free medium. The scratch was imaged with a microscope.

### Identification of candidate miRNAs targeting HOXC10

We used three miRNA prediction websites: Pictar (https://pictar.mdc-berlin.de/), Miranda and miRDB (http://mirdb.org/). The overlapping miRNAs were analysed.

### Luciferase reporter assays

HEK-293T cells were cultured and transfected with the psi-Check2-HOXC10 3’-UTR WT/psi-Check2 HOXC10 3’-UTR MUT plasmids in accordance with the Lipofectamine 2000 transfection system protocol. After incubation for 24 h, cells were lysed with 1× PLB and added to 96-well plates (Nunc™, Thermo Fisher Scientific, Denmark). Luciferase activity was assessed with a Dual-Luciferase® reporter assay kit (Promega, Madison, WI, USA). The luciferase activity signal ratio was calculated for each construct.

### ChIP-qPCR

We used JASPAR to identify the Slug binding sites in the HOXC10 promoter region. Chromatin was immunoprecipitated with an anti-HOXC10 antibody (ab153904; Abcam) and an IgG control antibody (ab2410; Abcam), and DNA was extracted and analysed with ChIP reagents (sc-45000, sc-45001, sc-45002, sc-45003; Santa Cruz Biotechnology) following the manufacturer's instructions. The primer sequences specific for the three predicted binding sites in the Slug promoter were as follows: site 1, 5’-TGGCGATATGTGTTTTCTCAACT-3’ (forward) and 5’-TGGAACCTGGAGTAAAAGCCA-3’ (reverse); site 2, 5’-CACCACATAAAAGCAGGGGAAT-3’ (forward) and 5’-GGTAACTGTCATTTGGAACCAC-3’ (reverse); and site 3, 5’-GCCTTTGTCTTCCCGCTTC-3’ (forward) and 5’-CCAGGAGAAGGAAGGGCC-3’ (reverse). The primer sequences specific for the nonpredicted binding site were as follows: 5’-CCCTCCTAGCTCCCAGAGAGAG-3’ (forward) and 5’-GGGACAGCTGGAACAGAGG- 3’ (reverse).

### IHC analysis

IHC staining was performed with an anti-HOXC10 antibody (1:400 dilution; DF9579; Affinity) and a visualization reagent. The tissue staining intensity and percentage were analysed with ImageJ software (version 1.51). According to the comprehensive score (cutoff value = 6), the staining intensity was scored as low (<5) or high (≥5). Morphological characteristics were observed and imaged under a microscope (Olympus Corp., Tokyo, Japan).

### Patients and samples

This study was approved by the ethics committee of Xiangya Hospital (Changsha, China). Written informed consent was obtained from the patients. All specimens were processed anonymously according to our ethics committee and investigational review board guidelines. Paraffin-embedded tissue samples and clinical information from 158 patients with OC were obtained from the pathology department of Xiangya Hospital between July 2010 and July 2015. Paraffin-embedded normal ovarian tissue samples from 10 patients were used as negative controls. Histological diagnosis and grading of tumours were performed in accordance with the 2009 FIGO staging guidelines (FIGO Committee and Working Group Publications) by at least two pathologists.

### Mouse xenograft model

This study was approved by the Central South University Institutional Animal Care and Use Committee for *in vivo* studies. All athymic nude mice (female, 4-6 weeks old) were purchased from and bred under pathogen-free conditions in the animal department of Central South University. Mice were randomly divided into three groups (five animals per group). Each group was injected intraperitoneally (i.p.) with 5×10^6^ cells. Mice in the OE-HOXC10 group were injected i.p. with both OC cells stably overexpressing the HOXC10 gene and the NC agomir (RiboBio Co., Ltd, Guangzhou, China). Mice in the NC group mice were i.p. OC cells that stably overexpressed vector and NC agomir. Agomir group mice were injected i.p. with both OC cells stably overexpressing the HOXC10 gene and the miR-222-3p agomir (RiboBio Co., Ltd, Guangzhou, China). The miR-222-3p agomir product was modified with an O-methyl moiety at the 2’-ribose position and the 5’ end in its terminal nucleotides at both ends; this agomir upregulated miR-222-3p expression in mice. The agomir products were injected directly into mouse enterocoelia at a dose of 1 nmol per mouse every 3 days for a total of twelve injections. Tumours were weighed after the mice were necropsied.

### Statistical analysis

All results in this study are presented as the mean±SEM values. Statistical significance was calculated by Student’s t-test. Two-way ANOVA was used for comparisons among multiple groups. All analyses were performed with GraphPad Prism 8 software (GraphPad Software, Inc., La Jolla, CA, USA).

## Supplementary Material

Supplementary Figures

Supplementary Tables
