# Supplementary material for: HOXC10 promotes tumour metastasis by regulating the EMT-related gene Slug in ovarian cancer
Source: Aging (Albany NY). 2020 Sep 7;12(19):19375–98. doi: 10.18632/aging.103824 (PMC7732328; doi:10.18632/aging.103824)
Supplement: Supplementary Figures [file aging-12-103824-s001..pdf]

SUPPLEMENTARY FIGURES

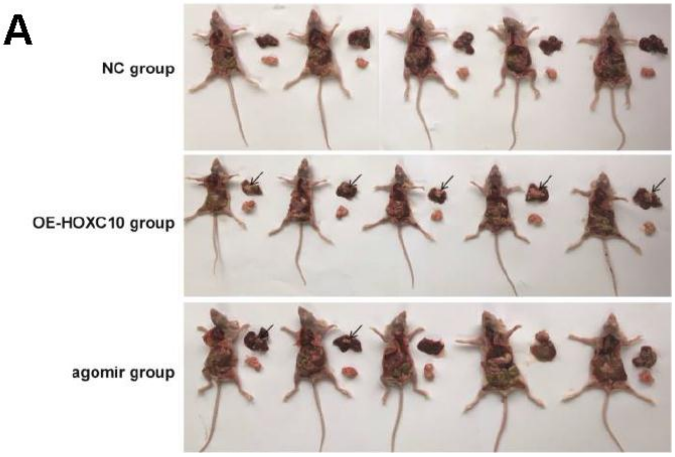

Supplementary Figure 1. Photograph of necropsied mice and excised tumours. (**A**) Picture presentation of mice after necropsy and intraperitoneal tumours were taken out.

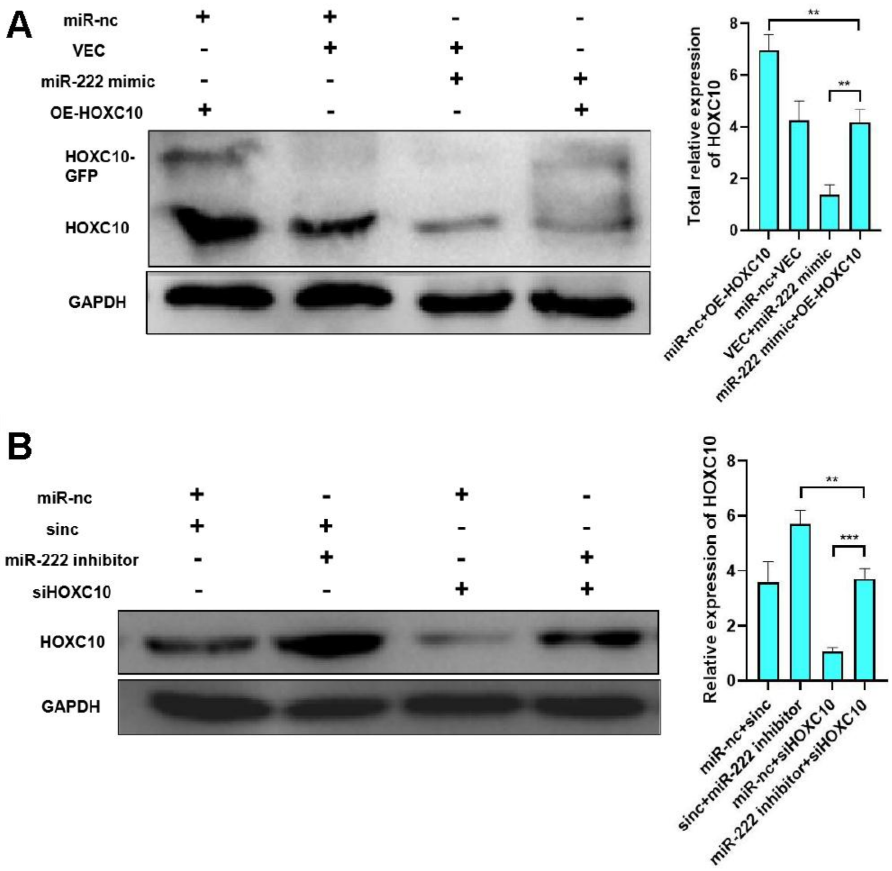

Supplementary Figure 2. HOXC10 protein expression level of miR-222-HOXC10 rescue experiment. (**A**) HOXC10 protein expression level of 8910 cell transfected with miR-222 mimic reagent and OE-HOXC10 plasmid. P=0.0037, P=0.0041. (**B**) HOXC10 protein expression level of 8910 cell transfected with miR-222 inhibitor reagent and siHOXC10 reagent. P=0.0053, P=0.0003.

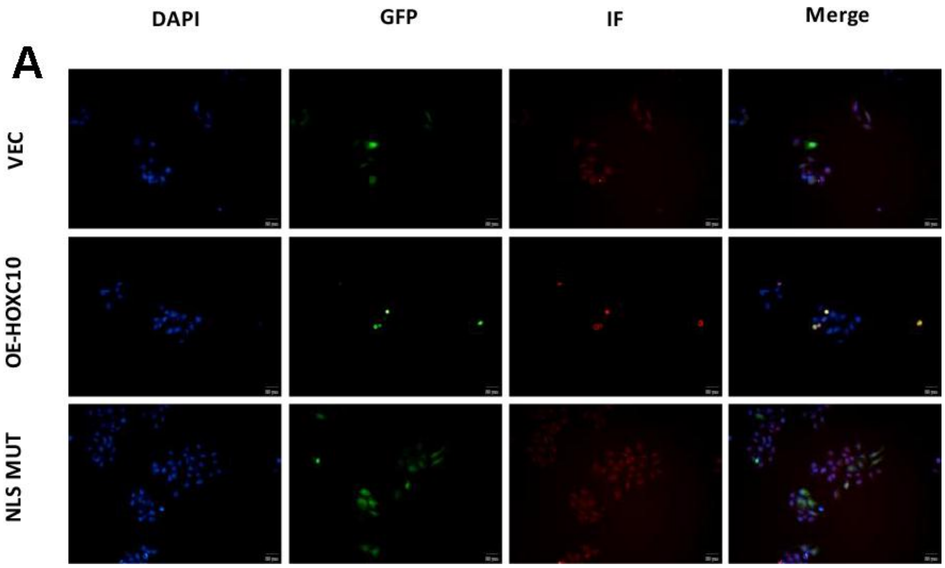

Supplementary Figure 3. Cell immunofluorescence staining of HOXC10. (**A**) Fluorescence signal in 8910 cells transfected with the HOXC10 overexpression plasmid, HOXC10 NLS mutation plasmid and empty vector. DAPI, blue. GFP, green. Cell immunofluorescence staining of HOXC10, red. Scale bars, 25 μm.

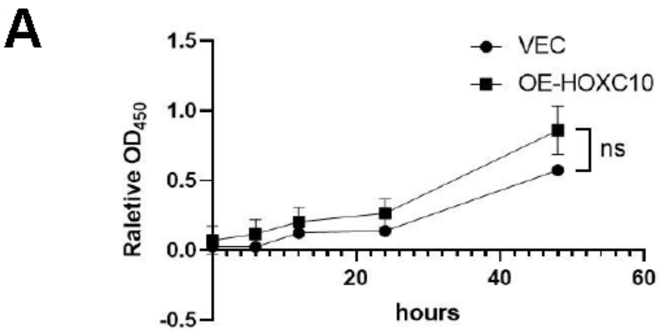

Supplementary Figure 4. Cell proliferation rate in HOXC10 stable cell model. (**A**) 8910 cell proliferation rate of transfected with VEC plasmid or OE-HOXC10 plasmid. P=0.4939.

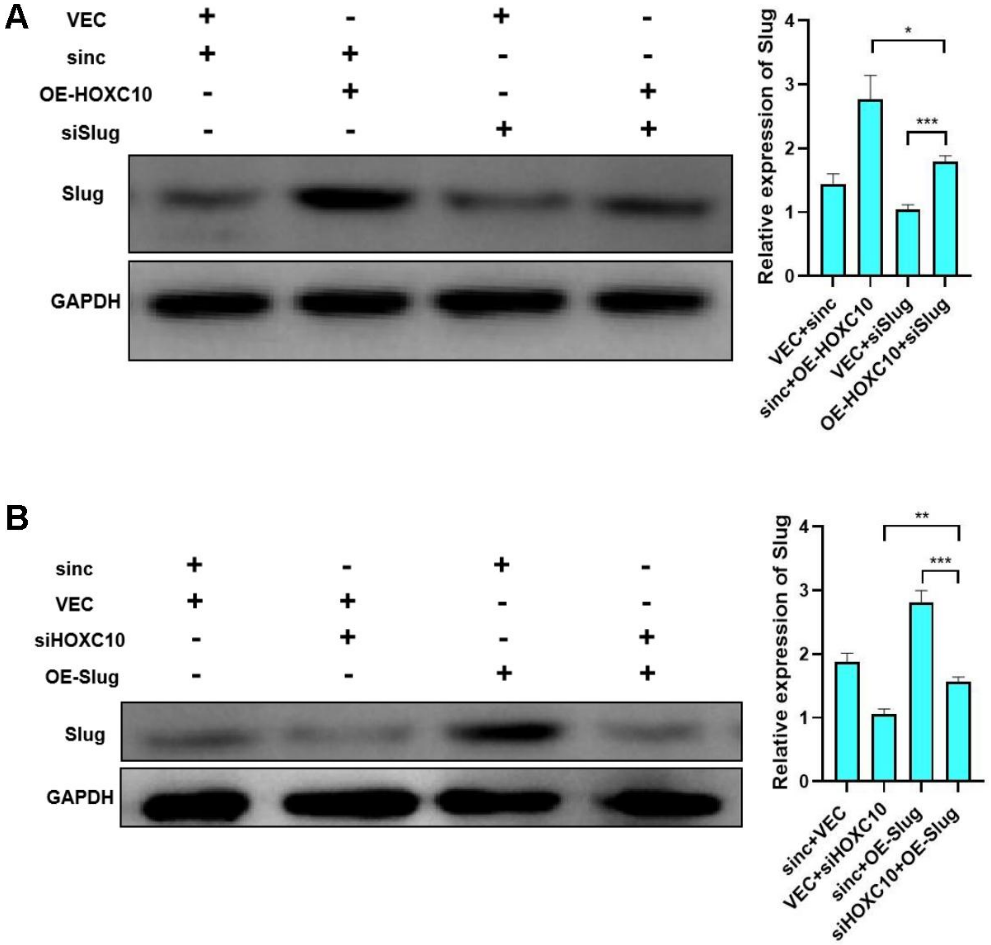

Supplementary Figure 5. HOXC10 protein expression level of HOXC10-Slug rescue experiment. (**A**) HOXC10 protein expression level of 8910 cell transfected with OE-HOXC10 plasmid and siSlug reagent. P=0.0121, P=0.0004. (**B**) HOXC10 protein expression level of 8910 cell transfected with siHOXC10 reagent and OE-Slug plasmid. P=0.0012, P=0.0005.

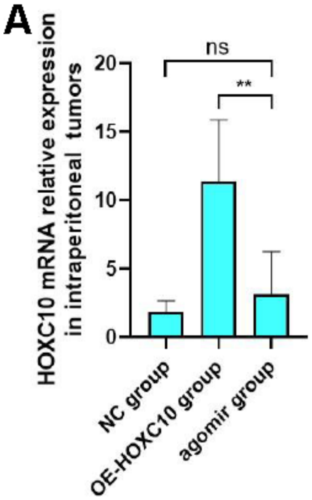

Supplementary Figure 6. HOXC10 mRNA expression level in mice intraperitoneal tumor tissues. (**A**) HOXC10 mRNA relative expression level in NC group, OE-HOXC10 group and agomir group mice intraperitoneal tumor tissues, P=0.4026, 0.0098.

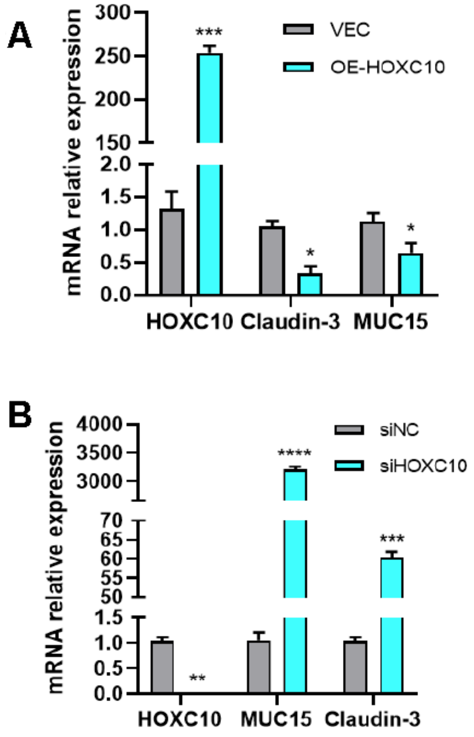

Supplementary Figure 7. EMT-related epithelial gene mRNA expression level. (**A**) Epithelial gene Claudin-3 and MUC15 mRNA expression level when cells up-regulated HOXC10 expression, P=0.0003, 0.0177, 0.0235. (**B**) Epithelial gene Claudin-3 and MUC15 mRNA expression level when cells down-regulated HOXC10 expression, P=0.0021, <0.0001, 0.0002.
