# Supplementary material for: HOXC10 promotes tumour metastasis by regulating the EMT-related gene Slug in ovarian cancer
Source: Aging (Albany NY). 2020 Sep 7;12(19):19375–98. doi: 10.18632/aging.103824 (PMC7732328; doi:10.18632/aging.103824)
Supplement: Supplementary Tables [file aging-12-103824-s002..pdf]

SUPPLEMENTARY TABLES

Supplementary Table 1. Univariate and multivariate analyses for overall survival of OC patients (N=158).

Variable	Univariate				Multivariate			
	P	HR	95%CI		P	HR	95%CI	
Age at diagnosis (<50 y vs. ≥50 y)	0.077	0.679	0.442	1.043	-	-	-	-
Histologic type (Serious vs. Nonserous)	0.062	0.656	0.422	1.022	-	-	-	-
FIGO Stage (I/II/III vs. IV)	0.011	0.627	0.437	0.901	0.03	0.663	0.458	0.961
Survival state (Alive vs. Dead)	0.004	0.532	0.346	0.819	0.009	0.557	0.358	0.865
Distant metastasis (Absence vs. presence)	0.013	0.633	0.441	0.909	0.018	0.639	0.442	0.926
HOXC10 level (low vs. high)	0.005	0.522	0.333	0.819	0.008	0.516	0.315	0.843

Samples: The Xiangya Hospital (Changsha, China); CI: confidence interval; HR: hazard ratio

Supplementary Table 2. Candidate microRNAs from three prediction websites.

Prediction websites	Candidate microRNAs						
miRDB	hsa-miR-7106-5p	hsa-miR-1299	hsa-miR-4739	hsa-miR-548x-5p	hsa-miR-4756-5p	hsa-miR-548aj-5p	hsa-miR-548g-5p
	hsa-miR-1321	hsa-miR-548f-5p	hsa-miR-4533	hsa-miR-136-5p	hsa-miR-4775	hsa-miR-12119	hsa-miR-875-3p
	hsa-miR-3148	hsa-miR-4733-3p	hsa-miR-9983-3p	hsa-miR-510-5p	hsa-miR-515-5p	hsa-miR-33a-5p	hsa-miR-519e-5p
	hsa-miR-33b-5p	hsa-miR-1908-5p	hsa-miR-10396b-5p	hsa-miR-663a	hsa-miR-129-5p	hsa-miR-6787-5p	hsa-miR-5706
	hsa-miR-4782-5p	hsa-miR-5011-3p	hsa-miR-766-5p	hsa-miR-6891-5p	hsa-miR-4441	hsa-miR-1251-3p	hsa-miR-516b-5p
	hsa-miR-3529-3p	hsa-miR-4436a	hsa-miR-5000-3p	hsa-miR-3173-3p	hsa-miR-12118	hsa-miR-4270	hsa-miR-6754-5p
	hsa-miR-1468-3p	hsa-miR-765	hsa-miR-11181-3p	hsa-miR-302b-5p	hsa-miR-4762-3p	hsa-miR-302d-5p	hsa-miR-7110-5p
	hsa-miR-7162-3p	hsa-miR-6799-5p	hsa-miR-6842-5p	hsa-miR-4749-5p	hsa-miR-4706	hsa-miR-329-5p	hsa-miR-10b-3p
	hsa-miR-6876-5p	hsa-miR-4476	hsa-miR-6878-5p	hsa-miR-6752-5p	hsa-miR-6068	hsa-miR-6780a-3p	hsa-miR-4689
	hsa-miR-3915	hsa-miR-6128	hsa-miR-4729	hsa-miR-4303	hsa-miR-5093	hsa-miR-4269	hsa-miR-6867-5p
	hsa-miR-6715b-5p	hsa-miR-5582-5p	hsa-miR-30c-2-3p	hsa-miR-30c-1-3p	hsa-miR-6788-5p	hsa-miR-362-3p	hsa-miR-329-3p
	hsa-miR-6780a-5p	hsa-miR-6828-5p	hsa-miR-3183	hsa-miR-1224-5p	hsa-miR-4673	hsa-miR-4326	hsa-miR-4645-5p
	hsa-miR-6780b-3p	hsa-miR-6805-3p	hsa-miR-5691	hsa-miR-6858-5p	hsa-miR-222-3p	hsa-miR-12120	hsa-miR-221-3p
	hsa-miR-6892-3p	hsa-miR-4780	hsa-miR-6824-3p	hsa-miR-6764-3p	hsa-miR-3679-5p	hsa-miR-3680-3p	hsa-miR-6769b-3p
	hsa-miR-3605-5p	hsa-miR-4779	hsa-miR-4518	hsa-miR-4521	hsa-miR-4723-3p	hsa-miR-1266-5p	hsa-miR-103a-3p
	hsa-miR-107	hsa-miR-7157-3p	hsa-miR-6737-3p	hsa-miR-3616-3p	hsa-miR-6079	hsa-miR-6785-5p	hsa-miR-4728-5p
	hsa-miR-1226-3p	hsa-miR-1185-5p	hsa-miR-8085	hsa-miR-5008-3p	hsa-miR-6731-5p	hsa-miR-6888-3p	hsa-miR-6811-3p
	hsa-miR-4474-5p	hsa-miR-6853-5p	hsa-miR-7161-5p	hsa-miR-149-3p	hsa-miR-1275	hsa-miR-3202	hsa-miR-6883-5p
	hsa-miR-5001-3p	hsa-miR-1911-3p	hsa-miR-6779-5p	hsa-miR-651-3p	hsa-miR-3689b-3p	hsa-miR-1273h-5p	hsa-miR-3689c
	hsa-miR-30b-3p	hsa-miR-3689a-3p					
miRanda	hsa-miR-296-3p	hsa-miR-760	hsa-miR-623	hsa-miR-574-5p	hsa-miR-641	hsa-miR-511	hsa-miR-220
	hsa-miR-129-5p	hsa-miR-329	hsa-miR-33a	hsa-miR-603	hsa-miR-662	hsa-miR-329	hsa-miR-362-3p
	hsa-miR-935	hsa-miR-603	hsa-miR-33b	hsa-miR-362-3p	hsa-miR-222-3p	has-miR-425	hsa-miR-28-3p
	hsa-miR-34c-3p	hsa-miR-514					
PicTar	hsa-miR-136	hsa-miR-129	hsa-miR-221-3p	hsa-miR-222-3p			
